# Altered metabolism and endoplasmic reticulum-mitochondria contacts in multiple sclerosis neurons

**DOI:** 10.3389/fneur.2026.1915238

**Published:** 2026-10-13

**Authors:** Celina Böse, Adriana Rehm, Zhixiao Wu, Anna-Sophia Karl, Sanja Augustyniak, Pia Renk, Anke Salmen, Ralf Gold, Konstanze F. Winklhofer, Barbara Gisevius, Verian Bader, Jeremias Motte

**Affiliations:** 1 Department of Neurology, St. Josef-Hospital Bochum, Ruhr University Bochum, Bochum, Germany; 2 Faculty of Medicine, Department for Biochemistry and Pathobiochemistry, Molecular Cell Biology, Ruhr University Bochum, Bochum, Germany

**Keywords:** endoplasmic reticulum, endoplasmic reticulum-mitochondria contacts, metabolism, mitochondria, multiple sclerosis

## Abstract

**Background:**

Genetic factors are estimated to account for approximately 30–50% of the risk for developing Multiple sclerosis (MS). Distinct mitochondrial variants, impairments in the respiratory chain, and mitochondrial dysfunction have been reported in individuals affected by MS. Mitochondria and the endoplasmic reticulum (ER) form membrane contact sites (ERMCs) that control important biological functions. Dysregulated ERMCs have been associated with a number of severe consequences for homeostasis, including altered mitochondrial calcium levels, impaired ATP production and abnormal lipid transfer. All of these mechanisms are thought to play an important role in the pathogenesis and disease progression of MS. Here, we investigated the hypothesis that the mitochondrial predisposition plays a crucial role in contributing to MS development.

**Methods:**

Mitochondrial functionality was analyzed in a model of human induced primary neurons (iPNs), and neuronal precursor cells (NPCs), differentiated from induced pluripotent stem cells (iPSCs) from people with MS and healthy controls (HCs). Seahorse analysis was performed for investigation of the bioenergetic profile of the cells. ERMC and mitochondrial morphological analysis were carried out to investigate the potential impact of mitochondrial dysfunction in MS-derived neurons.

**Results:**

Seahorse analysis revealed significant differences in the respiratory complexes (spare capacity: *p* = 0.0002; non-mitochondrial oxygen consumption: *p* = 0.0207) and glycolysis (*p* = 0.0001; glycolytic reserve *p* = 0.0018) from MS-derived iPNs compared to HCs. We further investigated mitochondrial dysfunction in NPCs (maximal respiration *p* = 0.0491; non-mitochondrial oxygen consumption: *p* = 0.0226) and a significant difference in glycolytic reserve (*p* = 0.0359) from MS-derived NPC compared to HCs. Furthermore, we identified a significantly higher amount of ERMCs (*p* < 0.0001) and altered mitochondrial morphology in MS-derived iPNs compared to HC.

**Discussion:**

Our data are highlighting the importance of genetic mitochondrial contribution to MS pathology, by showing that MS-derived NPCs and iPNs exhibit altered mitochondrial behavior and morphology compared to healthy control (HC)-derived cells.

## 1 Introduction

Multiple sclerosis (MS) is the most common chronic neuroinflammatory disease of the central nervous system (CNS) (1), characterized by CNS-invading autoreactive immune cells, causing demyelination and destruction of neurons, resulting in progressive neurodegeneration and irreversible neuronal loss (2–6). As a consequence, the energy demand of neurons increases in order to maintain the physiological ion homeostasis. Furthermore, the genetic contribution to the overall risk factors known to contribute to the development of MS can be estimated at 30–50% (7–9). Distinct variants of mitochondrial genetics were found to increase the risk of developing MS and are crucial for the biological function of each cell by, i.e., providing energy that is essential for cellular survival and metabolism (10, 11). In MS, mitochondrial dysfunction has been studied as a result of impaired and altered expression of respiratory chain complexes (12, 13). However, it has also been shown that mitochondrial oxidation occurs prior to the infiltration of inflammatory cells into the CNS and that mitochondria divide and are transported to demyelinated regions to maintain neuronal functionality (14–17).

In addition, mitochondria form membrane contacts with the endoplasmic reticulum (ER) and play an important role in ATP production, which is provided by oxidative phosphorylation (oxPhos), that is regulated by Ca^2+^ (18–23) and dysregulation of these ER-mitochondria contact sites (ERMCs) has been investigated in other neurodegenerative diseases (24, 25).

Therefore, altered energy demand plays an important role in the susceptibility and progression of MS, particularly through mitochondrial dysfunction and reactive oxygen species (ROS) production (12, 26, 27).

We hypothesize that there is evidence that a genetic predisposition of mitochondrial dysfunction in MS essentially contributes to the 30–50% of genetic risk factor that promotes disease onset and progression. Importantly, the induced pluripotent stem cell (iPSC) model is uniquely suited to dissect the intrinsic, genetic component of mitochondrial dysfunction in MS: during reprogramming of somatic cells to iPSCs, all acquired epigenetic modifications and environmental influences accumulated over the donor’s lifetime are erased (28, 29). Consequently, any phenotypic differences observed between MS- and HC-derived cells cannot be attributed to prior disease activity, medication, or lifestyle factors, but instead reflect the underlying genetic predisposition. Here, we analyzed the mitochondrial and glycolytic metabolism via Seahorse analysis in our iPSC-based neuronal model of human induced neuronal precursor cells (NPCs) and primary neurons (iPNs) differentiated from iPSCs of people with MS and healthy controls (HCs) (30, 31). Furthermore, differences in the mitochondrial network were analyzed by investigating mitochondrial morphology and the membrane contact sites of mitochondria and the ER.

## 2 Methods

### 2.1 Patients

This study was approved by the ethics committee of the Faculty of Medicine, Ruhr University Bochum (Reg. no. 4493–12), and the participants included into this study were affiliated with the Department of Neurology, St. Josef-Hospital, Bochum, University Hospital of the Ruhr-University Bochum, Germany. The study was performed in accordance with the ethical standards as laid down in the Declaration of Helsinki. Following written consent, 200 mL urine were collected from study participants. Informed consent was obtained from all individual participants included in the study.

Of people with MS included in this study (*N* = 2–3), two males (age 32) and one female (age 35), were diagnosed with relapsing–remitting multiple sclerosis with an expanded disability status scale (EDSS) of 2.0–3.0 at inclusion. After diagnosis the urine was collected in between 5 and 15 years of disease. All people with MS included in this study had a different disease-modifying therapy (Fingolimod, Natalizumab or Dimethyl fumarate; Supplementary Table 1). One female (age 25) and one male (age 29) were included as healthy controls in this study. At least one iPSC clone per donor was generated. Generation of iPNs and NPCs was performed as described previously (30, 31). Briefly, renal proximal tubule epithelial cells (RPTECs) were isolated by several centrifugation and washing steps and by resuspending in warmed proliferation medium (REGM™; Lonza) transferred into a cell culture plate. The medium was changed every second day. After reaching a confluency of around 90% (usually after 2–3 weeks) cells were trypsinized and plated on a cell culture dish. After passaging for three times, approximately 1 million RPTECs were harvested for transfection via electroporation with episomal vectors (hOCT3/4, hUL, hSK: Addgene plasmids #27077, #27078, #27080) to generate induced pluripotent stem cells (iPSCs). By reaching a confluency of 70% the medium was switched to a specific reprogramming medium (TeSR™-E7™; Stemcell Technolgies) and passaging of cells were performed every week. For embryoid body formation, iPSCs were cultured in a free-floating dish with mTeSR™ 1 medium, supplemented by SB431542 (Biozol) and dorsomorphin (Sigma) to suppress the meso-, epi- and entodermal lineages and induce the ectrodermal lineage. Medium change occurred every 2 days until day 6. Afterwards, the embryoid bodies were plated on a cell culture dish for adherent cultivation with neuronal stem cell (NSC) medium for neural rosette structure formation. During that time, the NSC medium was supplemented with insulin (Sigma, 91077C), B27™ (Thermo Scientific, #17504001), N-2 supplement (Thermo Scientific, #17502001) and epidermal growth factor (EGF, PAN Biotech, #1101001) as well as basic fibroblast growth factor (bFGF, PAN Biotech, #1102021). Neural rosettes were formed after approximately 7–12 days and isolated for cultivation to neuronal progenitor cells. For induction of induced primary neurons, medium of NPCs were changed and supplemented with Sonic Hedgehoc C25II protein (PeproTech, #100–45) and retinoic acid (Sigma, #R2625) for 7 days. Afterwards, the medium was supplemented with brain-derived neurotrophic factor (BDNF; PeproTech, #450–02) and glia-derived neurotrophic factor (GDNF; PeproTech, #450–10) onwards. On day 21 cells were harvested and used for experiments.

### 2.2 Seahorse analysis

Mitochondrial and glycolytic stress test were performed by using the Agilent Seahorse XF Cell Mito Stress test Kit (Agilent technologies, item no. 103015–100) and Agilent Seahorse XF Cell Glycolysis Stress Test Kit (Agilent technologies, item no. 103020–100) with the Seahorse Bioscience XF96 Extracellular Flux Analyzer (Agilent Technologies).

For mitochondrial respiration and glycolytic analysis of iPNs and NPCs, 200,000 cells/well were plated on a sterile 96-well plate, coated with 0.002% poly-L-ornithine (Sigma Aldrich, P4957-50ML) and 10 μg/mL laminin (Sigma Aldrich, L2020-1MG). Cells were incubated for 24 h in respective cell culture medium at 37 °C and 5% CO_2_ described by Massa et al., 2016. Both tests were performed according to manufacturer’s specifications. Prior to each experiment cell viability, attachment of the cells was assessed by brightfield microscopy. Non-adherent cells were removed by replacing the medium. Pilot experiments of 3 trails with MS- and HC-derived cells were conducted to assess cell viability (Supplementary Table 2).

The Seahorse medium for the Mito Stress test contains 1 mM sodium pyruvate (Agilent Technologies, 103,578–100), 10 mM glucose (Agilent Technologies, 103,577–100) and 2 mM -glutamine (Agilent Technologies, #103579–100) dissolved in DMEM/F12 (Agilent Technologies, #103575–100). The Seahorse medium for the Glycolysis Stress test contains 2 mM L-glutamine dissolved in DMEM/F12.

The sensor cartridge ports were loaded as follows:

Mito Stress test: medium: port A - ATP-synthase inhibitor oligomycin (2 μM), port B - uncoupling agent carbonyl cyanide-4 (trifluorometh oxy) phenylhydrazone (FCCP) (1 μM) which disrupts the mitochondrial membrane potential and port C - a mixture of 0.5 μM rotenone (complex I inhibitor) and antimycin A (complex III inhibitor).

Glycolytic Stress test: port A - 10 mM glucose, port B - 1 μM oligomycin and port - C 100 mM 2-Desoxyglucose (2-DG).

### 2.3 Transfection

To analyze ERMCs, cells were transfected with 4xmts-mNeonGreen (AddGene, 98,876)/TOM20-eGFP (modified by AddGene) and ER-mScarlet (AddGene, 137,805)/ mCherry-ER-3 (AddGene, 55,041) plasmids, using the tested and most effective Lipofectamine ™ 3,000 (Thermo Scientific, L3000001). Coverslips were coated with 0.002% poly-L-ornithine (Sigma Aldrich, P4957-50ML) and 10 μg/mL laminin (Sigma Aldrich, L2020-1MG). 150,000 cells of iPNs or 100,000 cells of NPCs (per well) were seeded in respective culture medium and incubated for 24 h at 37 °C and 5% CO_2_. The next day, medium was replaced with respective fresh medium. Afterwards, the transfection mix was prepared as described by the company and cells were incubated for a minimum of 3 and a maximum of 6 h depending on HC- or people with MS-derived cells at 37 °C and 5% CO_2_. The efficiency of transfection was monitored at regular intervals from 3 h on up to 6 h. Once transfected cells were observed the transfection medium was replaced by the cell culture medium and images were taken. Cells were mounted with DAPI-Fluoromount-G® (Southern Biotec, SBA-0100-20). Analysis of ERMCs was performed on Z-stack (9 stacks) images acquired with a 63x oil objective of a high-resolution Elyra 7 lattice-SIM Zeiss microscope using Leap mode resulting in 27 z-planes of 0.110 μm with a total stack of 2.96 μm after SIM processing. 3D reconstruction of the mitochondria and ER was performed using Imaris (Oxford Instruments) and Fiji software (National Institutes of Health). For 3D reconstruction transfected mitochondria and the ER were marked manually and the threshold was set based on the marked region. The contact area of organelles was analyzed using an Imaris MATLAB (MathWorks) plugin-“Surface-Surface contact area.” The percentage of contact sites were calculated via Excel (Microsoft®). For ERMC and mitochondrial morphological analysis 6 separate experiments were conducted with approximately 10 cells per individual cell line.

### 2.4 Mitochondrial morphology

For the analysis of mitochondrial morphology, transfection and 3D reconstruction were performed as described above. Mitochondrial networks represented as density per cell, average mitochondrial sphericity, area (μm^2^), volume (μm^3^) and ellipsoid length of X-, Y- and Z-axis (μm) per cell were calculated by Imaris. The average of these parameters was calculated via Excel (Microsoft®), the mitochondrial shape factor was analyzed as ratio of X- or Y-axis to Z-axis (length-to-width ratio). Analysis was performed as described above. Brief, same images as for ERMECs analysis were used. After the 3D reconstruction was performed in Imaris, All calculations were automatically analyzed by the Imaris surface module of the software.

### 2.5 Statistical statement

Experimental results are non normalized, unblinded and presented as mean ± SD with n being the number of technical replicates or single cells. Technical replicates are defined as independent experiments performed on different days and using different passages. In each experiment triplicates per donor-derived cell line was used and the mean of those triplicates or single cells, are represented as individual values/points. The biological replicates representing the number of different donor-cell lines (patient: 3 and healthy controls 2, in total 5). The samples of each individual human were always cultured and plated separately. For the graphical and statistical illustration the means, generated of each individuum, were taken together for either MS-derived or HC-derived representations (Supplementary Figure 1). Biological replicates are defined as “N” and each technical replicate is defined as “n.” The group sizes are mentioned in each figure legend. Data sets were first tested for normality using the D’Agostino-Pearson omnibus normality test with a confidence level of 95%. Statistical tests for parametric distributed data were performed with unpaired T-test and for non-parametric data with Mann–Whitney-U-Test. Data were considered significant when *p*-values referred to *p < 0.05. All tests were performed using GraphPad Prism 8 GraphPad by Dotmatics.

## 3 Results

### 3.1 Bioenergetic analysis with seahorse technology

#### 3.1.1 Altered mitochondrial phenotype in iPNs and NPCs differentiated from people with MS

Differences in mitochondrial functionality of iPNs and NPCs derived from MS and HCs were analyzed via Seahorse Mito Stress Test. Mitochondria in iPNs derived from HCs showed no overall higher oxygen consumption rate than those derived from MS (Two-way ANOVA: *F* (1, 14) = 0.9890, *p* = 0.3369; Figure 1A). There was no significant difference in basal and maximal respiration, proton leak and ATP production between HC- and MS-derived iPN mitochondria (Figure 1B). However, we observed a significant decrease in both non-mitochondrial oxygen consumption (*U =* 10, *p* = 0.0207) and spare capacity (*t* (14) *=* 5.11, *p* = 0.0002) in MS-derived iPNs compared to HC-derived iPNs (Figure 1B).

**Figure 1:**
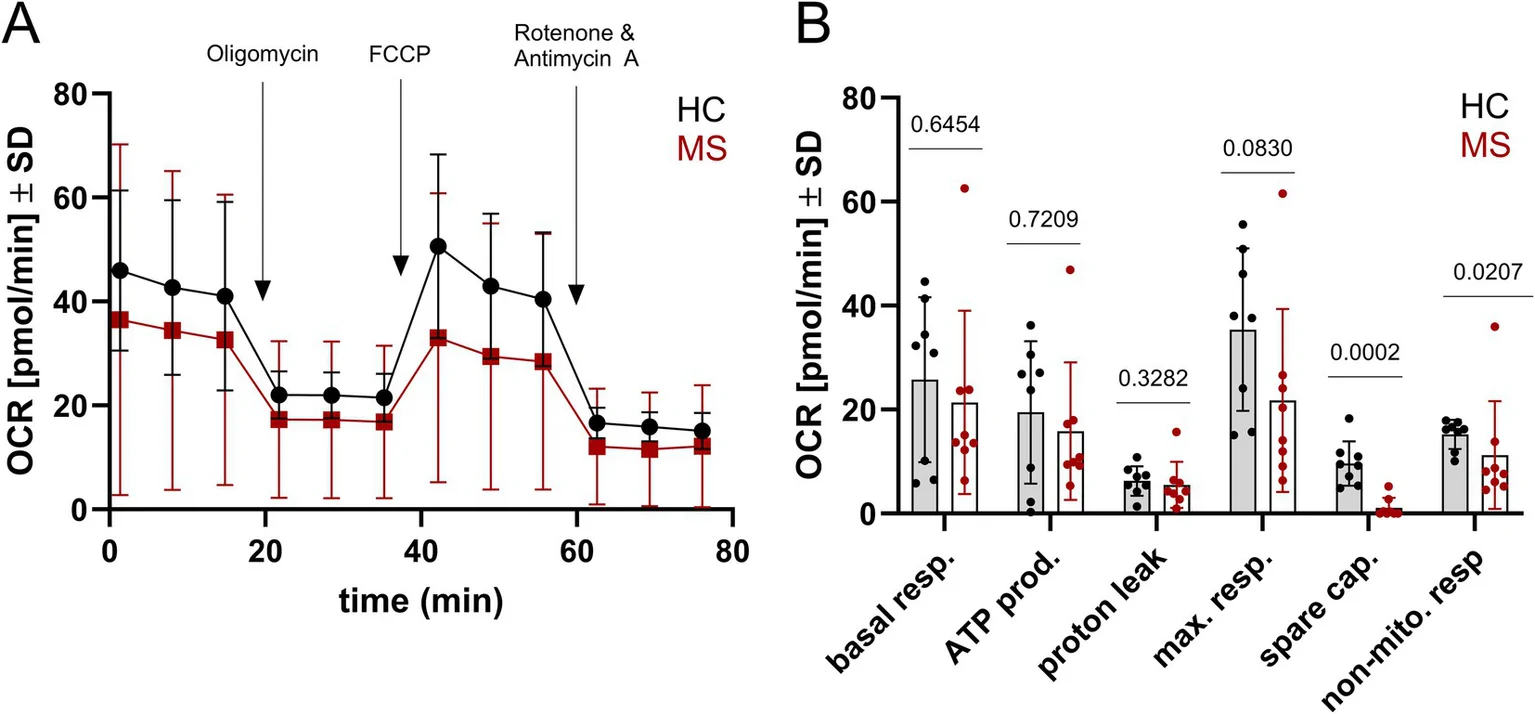
Data summary of Seahorse Mito-Stress Test assay from iPNs. **(A)** Mito-Stress Test data for OCR of MS- and HC-derived iPNs in pmol/min. Arrows indicate the injections of specific stressors oligomycin, FCCP and rotenone/antimycin A into media. **(B)** Relative values of mitochondrial respiration parameters including basal respiration, ATP production, proton leak, maximal respiration, spare capacity and non-mitochondrial respiration compared between MS- and HC-derived iPNs. Significant changes between MS- and HC-derived iPNs were observed in spare capacity and non-mitochondrial respiration. Data represented as mean ± SD; *N*(MS) = 3, N(HC) = 2; *n* = 8; unpaired *t* test or Mann–Whitney test. OCR, Oxygen consumption rate; FCCP, Carbonyl cyanite-4 (trifluoromethoxy) phenylhydrazone; ATP, Adenosine triphosphate; pmol, pico mol.

Mitochondria in NPCs derived from HCs showed a higher oxygen consumption rate compared to mitochondria in NPCs differentiated from MS (Two-way ANOVA: *F* (1.14) = 5.644, *p =* 0.0323; Figure 2A). A significant reduction in the maximal respiration (*t* (14) *=* 2.155, *p* = 0.0491) and non-mitochondrial oxygen consumption (*t* (14) *=* 2.561, *p* = 0.0226) was observed in mitochondria from MS-derived NPCs compared to mitochondria from NPCs differentiated from HCs (Figure 2B).

**Figure 2:**
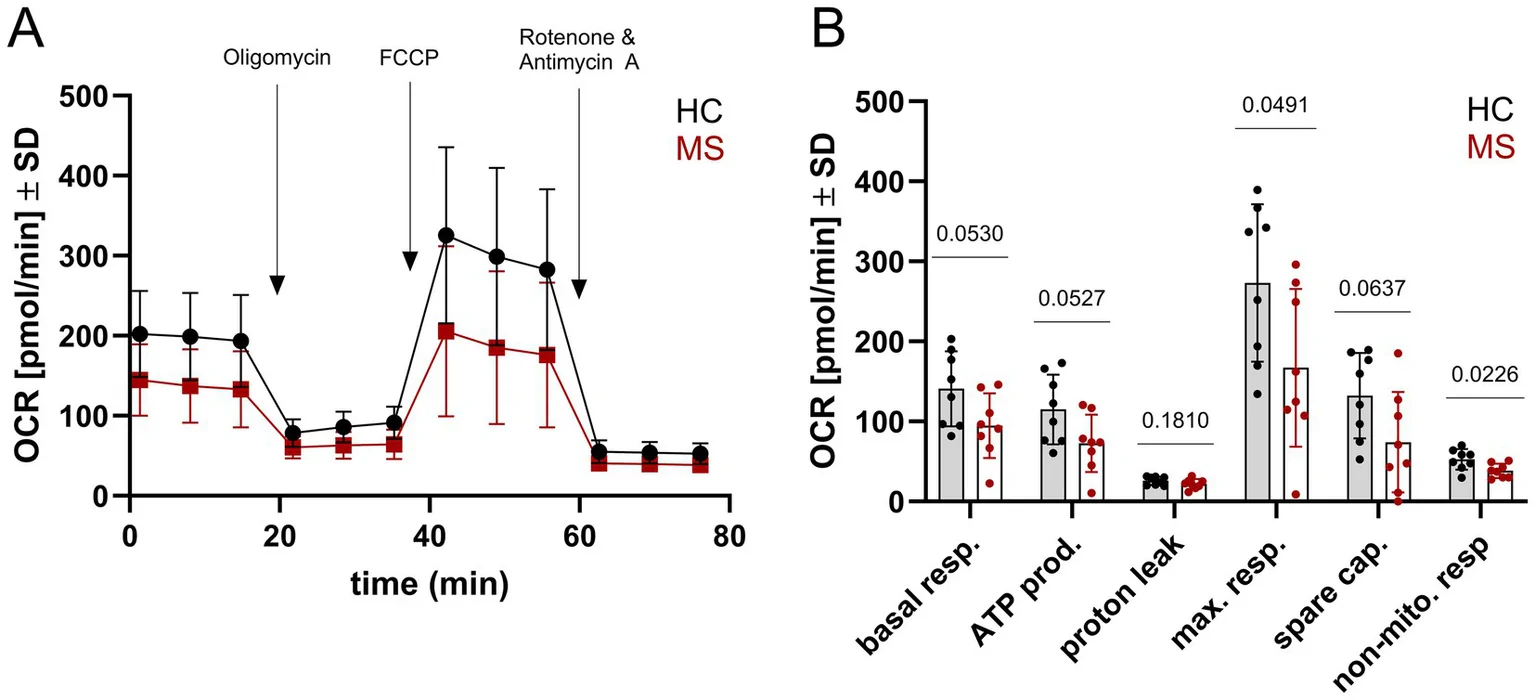
Data summary of Seahorse Mito-Stress Test assay from NPCs. **(A)** Measurement of the OCR of NPCs differentiated from MS and HCs in pmol/min. Arrows indicate the injections of specific stressors oligomycin, FCCP and rotenone/antimycin A into media. **(B)** Relative values of mitochondrial respiration parameters including basal respiration, ATP production, proton leak, maximal respiration, spare capacity and non-mitochondrial respiration compared between MS- and HC-derived NPCs. Significant changes between MS- and HC-derived NPCs were observed in maximal respiration and non-mitochondrial respiration. Data represented as mean ± SD; N(MS) = 2, N(HC) = 2; *n* = 8, unpaired t test or Mann–Whitney test. OCR, Oxygen consumption rate; FCCP, Carbonyl cyanite-4 (trifluoromethoxy) phenylhydrazone; ATP, Adenosine triphosphate; pmol, pico mol.

In summary, NPCs derived from MS showed a mitochondrial alteration compared to mitochondria in HCs. MS-derived NPCs showed a significant decrease in maximal respiration and non-mitochondrial oxygen consumption compared to HC-derived NPCs, similar to iPNs.

#### 3.1.2 Altered glycolysis in iPNs and NPCs differentiated from MS

Based on the previously observed alterations in mitochondrial respiration in MS-derived compared to HC-derived cells, we hypothesized that cellular glycolysis might also be affected. To investigate this, glycolytic function was analyzed using extracellular acidification rate (ECAR) measurements. ECAR was significantly higher in HC-derived iPNs compared to MS-derived iPNs (Two-way ANOVA: *F* (1, 24) = 46.41, *p* < 0.0001; Figure 3A). Non-glycolytic acidification was markedly reduced in iPNs differentiated from people with MS compared to those from HCs (*t* (25) *=* 7.317, *p* < 0.0001). Moreover, glucose stimulated glycolytic activity (*t* (25) *=* 4.619, *p* = 0.0001), glycolytic capacity (*U =* 10, *p* < 0.0001) and glycolytic reserve (*U =* 29, *p* = 0.0018) were significantly decreased in MS-derived iPNs in contrast to HC-derived iPNs (Figure 3B).

**Figure 3:**
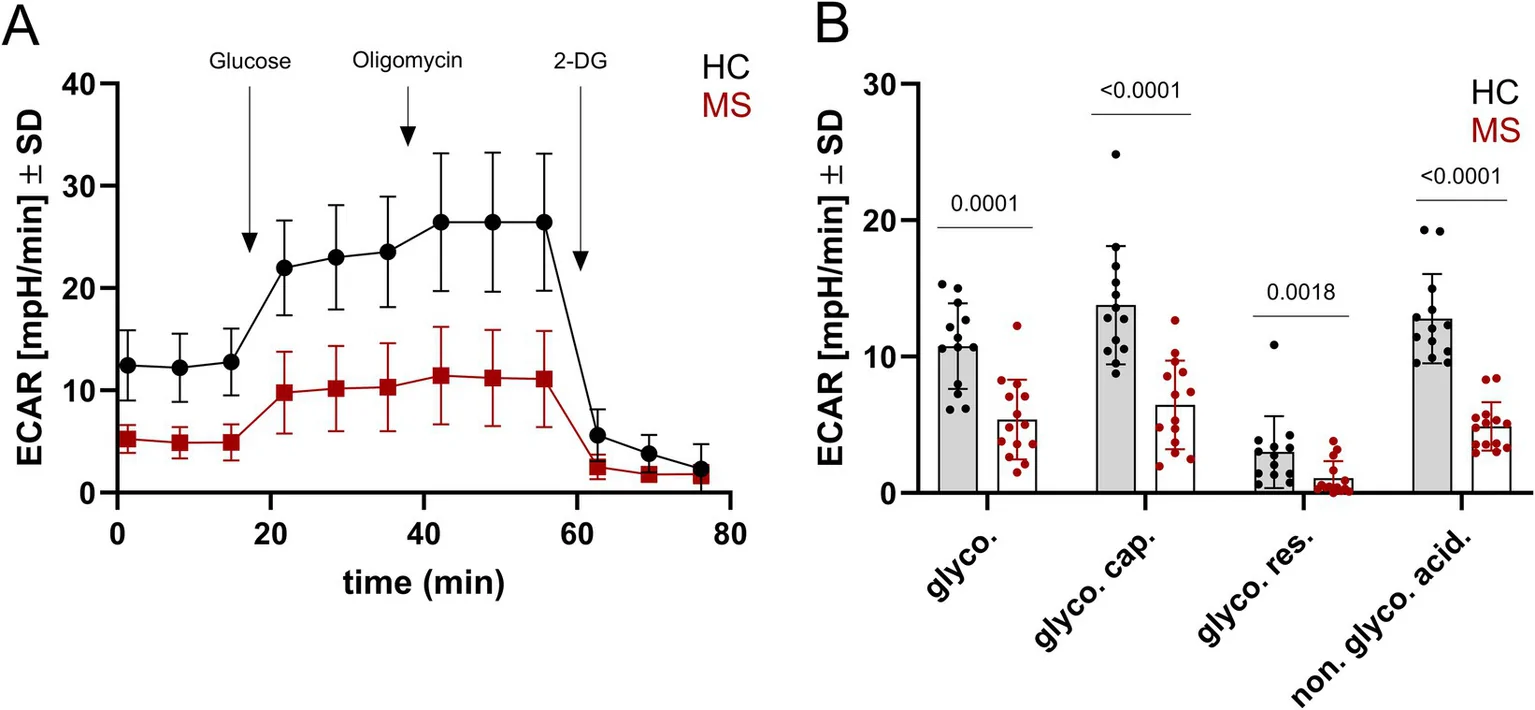
Data summary of Seahorse glycolysis stress assay from iPNs. **(A)** Measurement of the ECAR of iPNs differentiated from HCs and MS in mpH/min. Arrows indicate the injection of glucose, oligomycin and 2-DG. **(B)** Relative values of glycolysis flux analysis including glycolysis, glycolysis capacity, glycolytic reserve and non-glycolytic acidification rate were compared between MS- and HC-derived iPNs. Every analyzed parameter of glycolysis flux was significantly decreased in MS-derived iPNs compared to HC. Data represented as mean ± SD; N(MS) = 2, N(HC) = 2; n(HC) = 13, n(MS) = 14; Mann–Whitney test ECAR, Extracellular acidification rate; mpH, milli pH units; DG- 2-desoxy-D-glucose.

To further explore whether glycolytic functionality is may impaired at an earlier stage, the Seahorse Glycolysis Stress test was performed in NPCs differentiated from people with MS and HCs (Figure 4). NPCs generally exhibited a higher extracellular acidification rate compared to iPNs (Two-way ANOVA: (HC): *F* (1, 21) = 110.3, *p* < 0.0001; (pwMS): *F* (1, 21) = 148.4, *p <* 0.0001; Figures 3A, 4A). Analysis of glycolysis showed no significant difference in MS-derived NPCs compared to HC-derived NPCs (Two-way ANOVA: *F* (1, 18) = 0.8021, *p* = 0.3832; Figure 4B). Similarly, no differences in maximal glycolytic capacity was observed between the two groups (Figure 4B). Interestingly, a significantly reduced glycolytic reserve (*t* (18) *=* 2.268, *p* = 0.0359) was found in MS-derived NPCs compared to HC. However, there was no difference in the rate of non-glycolytic acidification rates between MS- and HC-derived NPCs (Figure 4B).

**Figure 4:**
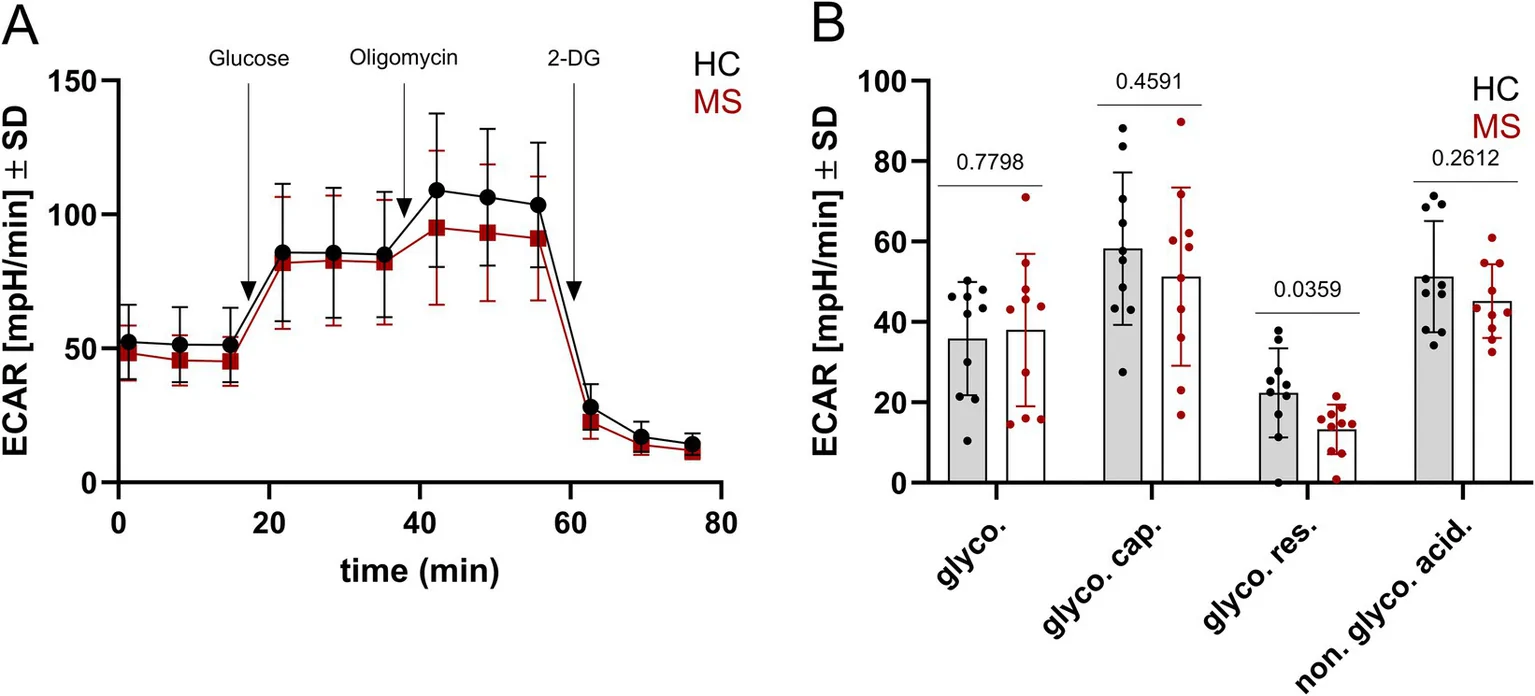
Altered glycolytic functionality observed in NPCs derived from MS in contrast to HCs. **(A)** Measurement of the ECAR of NPCs differentiated from HCs and MS in mpH/min. Arrows indicate the injection of glucose, oligomycin and 2-DG. **(B)** Relative values of glycolysis flux analysis including glycolysis, glycolysis capacity, glycolytic reserve and non-glycolytic acidification rate were compared between MS- and HC-derived NPCs. Glycolytic reserve was significantly decreased in MS-derived NPCs compared to HC. Data represented as mean ± SD; *N*(MS) = 3, N(HC) = 2; *n* = 10; unpaired t test or Mann–Whitney test. ECAR, Extracellular acidification rate; mpH, milli pH units; DG- 2-desoxy-D-glucose.

Overall, these findings demonstrate a significant impairment in glycolytic function in MS-derived iPNs compared to HC-derived iPNs, whereas only the glycolytic reserve appeared affected in the corresponding NPCs.

### 3.2 Analysis of ERMC

#### 3.2.1 Alterations of ERMCs observed in MS-derived iPNs compared to HC-derived iPNs but not in respective NPCs

Given the previously observed differences in mitochondrial respiration and glycolysis we examined ERMCs, crucial for metabolic coupling and important for mitochondrial respiration. Fluorescent images of HC- and MS-derived iPNs (Figure 5A) and NPCs (Figure 5E) were reconstructed into a 3D model (Figures 5B,F) and the contacts between the ER and mitochondria were measured (Figures 5C,G). The contact areas were calculated relative to the total mitochondrial area. For calculated contact areas normalized to total mitochondrial area, ERMCs were significantly higher in MS-derived iPNs (*U =* 1,259, *p* < 0.0001), compared to those from HCs (Figure 5D). To analyze whether changes in ERMCs are already present prior to the differentiation of NPCs into iPNs, ERMCs in NPCs were further analyzed. Although, compared to iPNs the average percentage of ERMCs was higher in NPCs, no significant difference was observed between MS- and HC-derived at the NPC stage regarding the ERMCs referenced to the total area of mitochondria (Figure 5H).

**Figure 5:**
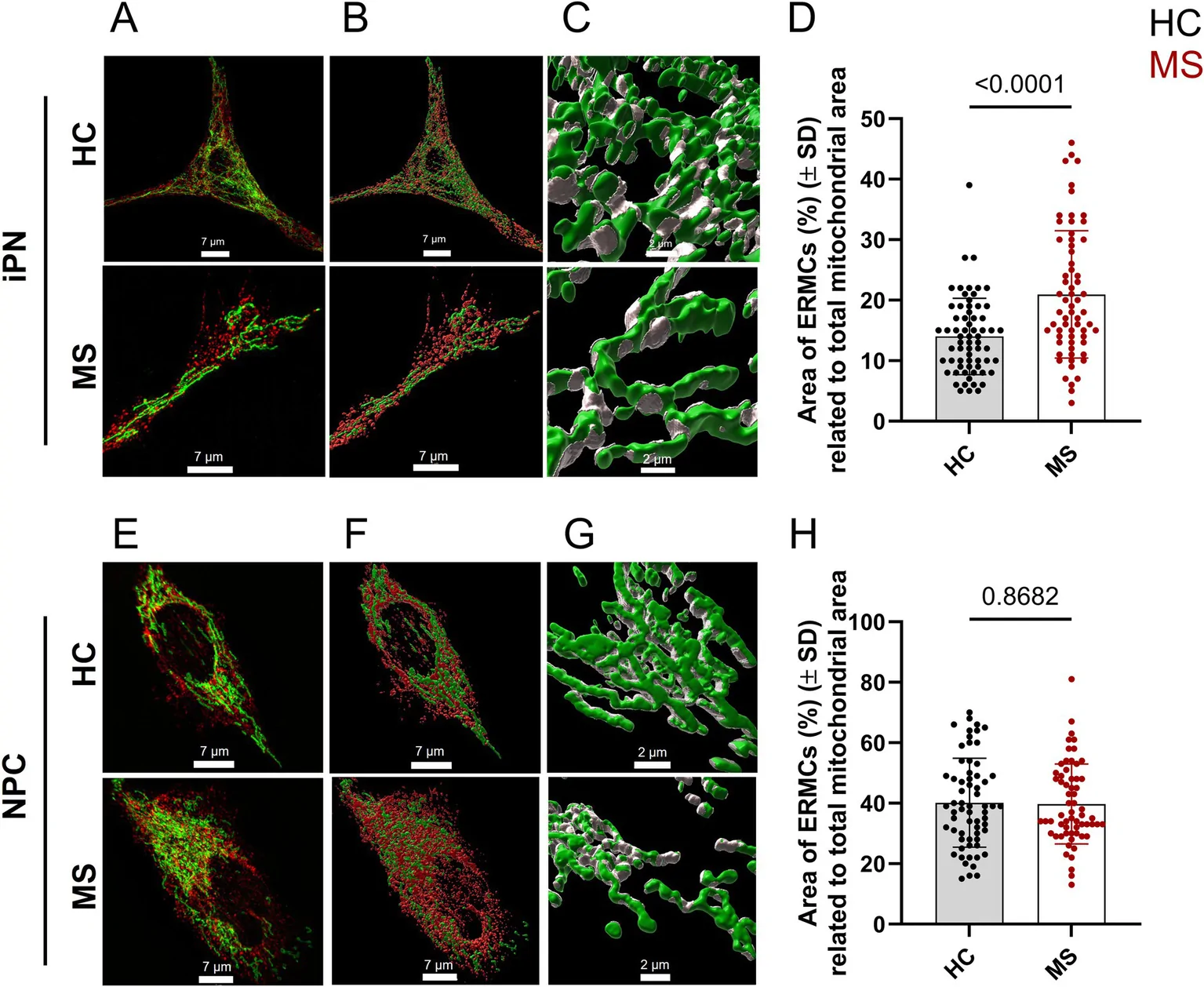
Summary of analyzed data of ERMC analysis with representative images of ERMC staining and analytic procedure of iPNs and NPCs. The images showed ER in red and the mitochondria in green. **(A)** Single cell fluorescence image of HC-derived (above) and MS-derived iPN (below) (Scale bar: 7 μm). **(B)** 3D reconstruction of a HC-derived iPN (above) and a MS-derived iPN (below) (Scale bar: 7 μm). **(C)** The grey areas represent the membrane contacts between the ER and mitochondria (HC-derived iPN above and MS-derived iPN below). The 3D reconstruction of the ER has been removed for clarity (Scale bar: 2 μm). **(D)** Percentage area of ERMCs related to total mitochondrial area was significantly higher in iPNs derived from MS compared to those from HCs. **(E)** Single cell fluorescence image of HC-derived (above) and MS-derived NPC (below) (Scale bar: 7 μm). **(F)** 3D reconstruction of a HC-derived NPC (above) and a MS-derived NPC (below) (Scale bar: 7 μm). **(G)** Grey areas represent the membrane contacts between the ER and mitochondria (HC-derived NPC above and MS-derived NPC below) (Scale bar: 2 μm). **(H)** Percentage area of ERMCs related to total mitochondrial area showed no significant differences in NPCs derived from MS compared to those from HCs. Data represented as mean ± SD; (D: *N*(MS) = 3, N(HC) = 2; *n*(HC) = 66, *n*(MS) = 64) (E: *N*(MS) = 3, *N*(HC) = 2, *n*(HC) = 64, *n*(MS) = 62); Mann–Whitney test; unpaired t test.

### 3.3 Analysis of mitochondrial morphology

#### 3.3.1 Altered mitochondrial morphology observed in iPNs and NPCs differentiated from MS and HCs

Since iPNs and NPCs differentiated from people with MS displayed differences in mitochondrial respiration, glycolysis and ERMCs, we further investigated whether morphological alterations of mitochondria are already present at the NPC stage prior to differentiation into iPNs. First, mitochondrial morphology parameters of MS- and HCs-derived iPNs were analyzed (Figure 6). A higher sphericity (*U =* 1,050, *p* < 0.0001) of mitochondria was observed in iPNs differentiated from HCs compared to iPNs differentiated from MS (Figure 6A). Furthermore, the mitochondrial area (Figure 6B) and volume (Figure 6D) were higher in iPNs differentiated from people with MS in comparison to iPNs differentiated from HCs (*U =* 1,060*, p* < 0.0001). However, the formation of mitochondrial networks per cell, was similar in iPNs from both groups, HCs and MS (Figure 6C). The analysis of the length and length-to-width ratio showed an increase in iPNs differentiated from people with MS compared to iPNs differentiated from HCs (Figure 6E). In summary, mitochondria in MS-derived iPNs displayed an increased mitochondrial area, volume, length, and length-to-width ratio, whereas the mitochondrial sphericity were decreased compared to mitochondria in HC-derived iPNs.

**Figure 6:**
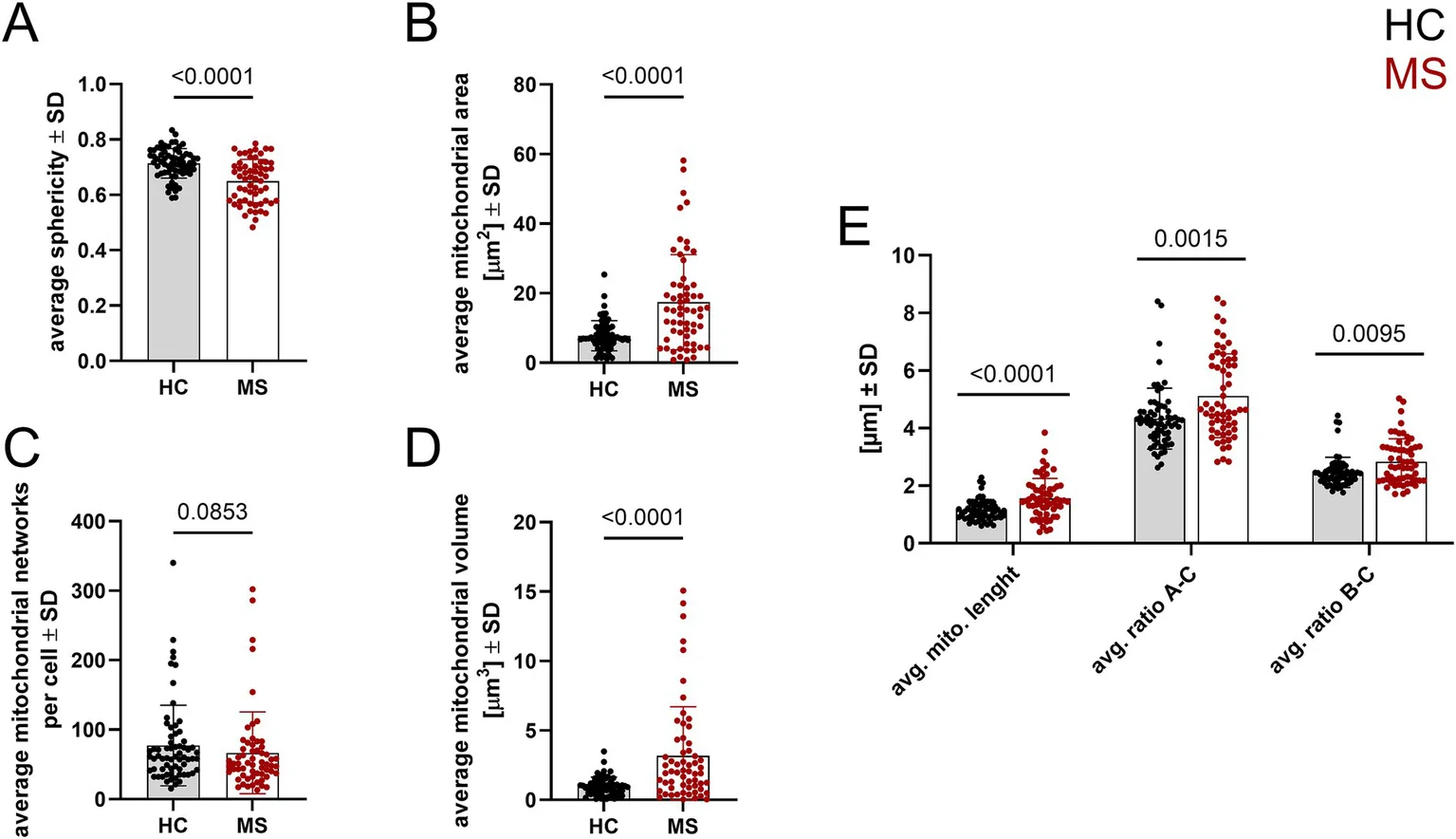
Altered mitochondria observed in iPNs differentiated from MS compared to HCs. **(A)** The sphericity represents the roundness with values of 1 indicating a perfect spheroid. Mitochondria in iPNs derived from HCs displayed a higher sphericity than in MS-derived iPNs. **(B)** In MS-derived iPNs a higher mitochondrial area was observed compared to HC-derived iPNs. **(C)** No difference in the amount of mitochondrial networks was investigated in iPNs derived from HCs and MS. **(D)** A higher mitochondrial volume was observed in iPNs differentiated from MS in comparison to HC-derived iPNs. **(E)** The length C and aspect ratio of the length C and width A and B was higher in mitochondria in MS-derived iPNs compared to HC-derived iPNs. Data represented as mean of each measurement per cell ± SD; *N*(MS) = 3, *N*(HC) = 2; *n*(HC) = 66, *n*(MS) = 60, Mann–Whitney test. Avg, average.

In contrast to the results in iPNs, mitochondria in MS-derived NPCs showed a marginally higher sphericity (*t* (124) = 4.067, *p* < 0.0001) compared to NPCs derived from HCs (Figure 7A). Furthermore, the mitochondrial area (*U =* 1,430, *p* = 0.0066) and volume (*U =* 1,491, *p* = 0.0159) were decreased in MS-derived NPCs in comparison to NPCs differentiated from HCs (Figures 7B,D). The number of mitochondrial networks (*U =* 1,042, *p* < 0.0001) was higher in MS-derived NPCs in contrast to NPCs differentiated from HCs (Figure 7C). Additionally, the average mitochondrial length (*U =* 1,289*, p =* 0.0006) as well as the length-to-width ratio of C_length_ to A_width_ (*U =* 1,328, *p =* 0.0012) in mitochondria was decreased in MS-derived NPCs in contrast to HC-derived NPCs. Interestingly, the length-to-width ratio of mitochondrial C_length_ to B_width_ in NPCs was not changed between both groups, MS and HCs (*U =* 1753, *p* = 0.2617; Figure 7E).

**Figure 7:**
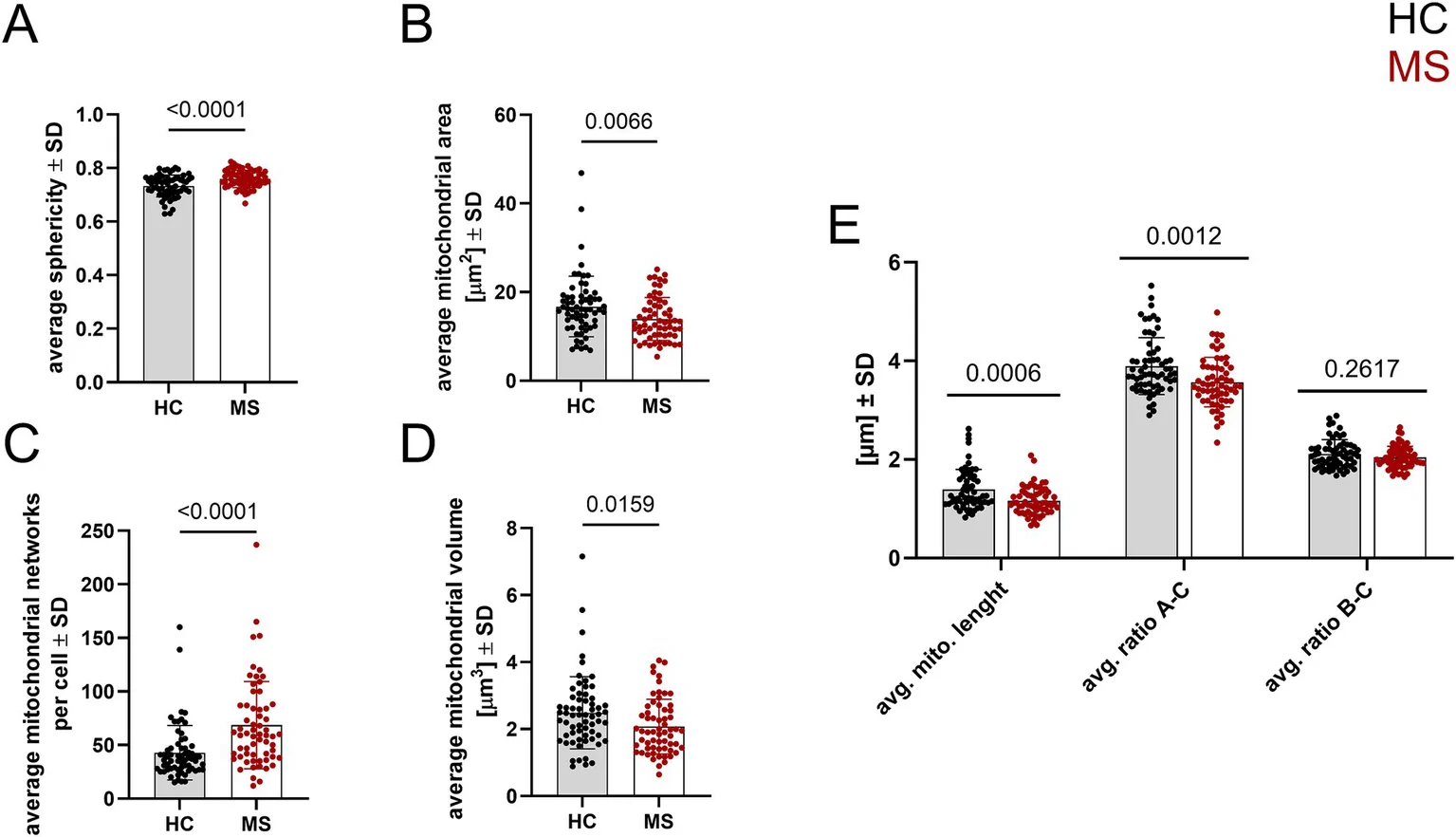
Altered mitochondrial morphology investigated in MS-derived NPCs compared to HCs. **(A)** The sphericity of mitochondria represents the roundness with a value of 1 indicating a perfect spheroid. Mitochondria in MS-derived NPCs displayed a higher sphericity in contrast to mitochondria in HC-derived NPCs. **(B)** The mitochondrial area was decreased in MS-derived NPCs in contrast to HC-derived NPCs. **(C)** Average mitochondrial density per cell was increased in MS-derived NPCs in comparison to those from HCs. **(D)** The mitochondrial volume was lower in MS-derived NPCs compared to NPCs differentiated from HCs. **(E)** Calculated length-to-width ratio of length C and width A showed a decreased aspect ratio in mitochondria in NPCs derived from MS compared to those from HCs. Calculated length-to-width ratio of length C and width B showed no difference in NPCs differentiated from MS in comparison to NPCs differentiated from HCs. The mitochondrial length in MS-derived NPCs displayed a decreased length compared to those from HCs. Data represented as mean ± SD; *N*(MS) = 3, *N*(HC) = 2; *n*(HC) = 64, *n*(MS) = 62, unpaired *t* test or Mann–Whitney test. Avg, average.

Altogether alterations in mitochondrial morphology were identified in MS compared to HCs both in iPNs and NPC.

## 4 Discussion

In this study, we used an iPSC-based neuronal model by reprogramming somatic cells of people with MS and HCs to induced pluripotent stem cells and differentiated them to NPCs and further iPNs as previously described (30, 31). This model was used to test our hypothesis of pre-existing mitochondrial dysfunction as well as whether a genetic predisposition for mitochondrial dysfunction plays a crucial role in MS pathogenesis, as the reprogramming eliminates the cell specific identity but retains the genetic burden of the patient (28, 29). Crucially, the complete epigenetic reprogramming inherent to iPSC generation largly erases (disease-related) epigenetic marks, environmental exposures, and medication effects that the donor has accumulated over their lifetime (32, 33). Therefore, any mitochondrial phenotype that persists or re-emerges after reprogramming and subsequent neuronal differentiation must be attributed to the genetic background rather than to acquired factors. This positions our model as an approach to investigate the genetic predisposition for mitochondrial vulnerability in MS, independent of confounding environmental influences. Here, we show alterations in mitochondrial function, mitochondrial morphology, and ERMCs in MS-derived iPNs and NPCs in comparison to HCs.

A decrease in the maximal respiration and spare capacity was identified in iPNs and NPCs derived from MS compared to HC. Both parameters are indicators for cell fitness or flexibility, representative for the respond to an increased energetic demand in MS neurons (34, 35). Our results indicate that mitochondria in MS are overall less flexible and viable to respond to this energetic demand, supporting the findings of dysfunctional or impaired mitochondria in MS, which could be caused by decreased complex (I/III) activity in the respiratory chain or mtDNA mutations, as previously observed in MS as well as in the animal model, the experimental autoimmune encephalomyelitis (EAE) (36–39).

In addition, the non-mitochondrial oxygen consumption was overall reduced in MS compared to HC, leading to the assumption that in MS most of the oxygen is used for energy production by oxidative phosphorylation (oxPhos). Conversely, the reduced non-mitochondrial oxygen consumption in MS-derived cells may indicate a lower activity of extra-mitochondrial oxygen-consuming enzymes such as NADPH oxidases, which could reflect an overall decrease in cellular metabolic capacity rather than a selective mitochondrial defect. No differences of oxygen consumption were observed under basal conditions as the inner mitochondrial membrane seems to be intact, indicated by a similar proton leak and ATP-production (34, 35).

As a consequence of the impaired functionality of the mitochondrial respiratory chain, we next analyzed whether energy is provided by glycolysis. In MS-derived iPNs, all glycolytic parameters were reduced compared to HC-derived iPNs, indicative of an impaired glycolytic functionality. However, in MS-derived NPCs, neither the non-glycolytic acidification nor the glycolysis, and glycolytic capacity showed differences between the two groups, suggesting that the glycolytic impairment occurs during the process of differentiation. Nevertheless, compared to HCs, the glycolytic reserve was significantly reduced in NPCs differentiated from people with MS, which leads to the assumption that the maximal capacity of these cells is close to their basal glycolysis and the cells were less flexible to respond to an energetic demand by glycolysis (34). It has also been reported that the maintenance of glycolytic metabolism in NPCs prevents oxidative damage and functions as protective mechanism (40). This supports our findings of similar glycolytic parameters in MS-derived NPCs but impaired mitochondrial functionality.

Comparing the mitochondrial and glycolytic functionality in NPCs and iPNs between MS and HCs, analysis of the glycolysis and respiratory chain revealed a higher ECAR and OCR in NPCs compared to iPNs. This is supported by the finding that induced neurons have a reduced oxPhos with aging, resulting in a lower OCR in iPNs than in NPCs (41, 42) as well as glycolysis being the main energy-producing pathway in NPCs (43–45). In addition, our data show that iPNs derived from people with MS are less resilient as the respective NPCs, as glycolytic parameters have changed more dramatically upon differentiation eventually due to energy demand, metabolic changes, and acquired mtDNA damage, which may impair the electron-transport-chain efficiency (36).

As mitochondrial functionality and biogenesis are also regulated by ERMCs and mitochondrial morphology (22, 46–49), we analyzed these parameters in more detail. The examined ERMCs were increased in MS-derived iPNs compared to HC-derived iPNs, but no difference was observed in NPCs. Interestingly, there were also contrary findings in mitochondrial morphology of NPCs and iPNs. Mitochondria in MS-derived iPNs displayed a lower sphericity and tend to a decreased number of mitochondrial networks, whereas the mitochondrial area, volume, and length were increased, assuming that the mitochondria were more fused (50, 51). In contrast, MS-derived NPCs exhibited a higher mitochondrial sphericity and number of mitochondrial networks compared to HC-derived NPCs. In addition, a decreased mitochondrial area, volume, and length were observed in MS-derived NPCs, indicating a more fragmented mitochondrial morphology compared to mitochondria in HC-derived NPCs (50, 51).

However, NPCs are proliferative and mainly glycolytic, while during differentiation a metabolic shift occurs, leading to non-proliferative iPNs, that are mainly oxPhos depending (40, 44, 45, 52). Therefore, we hypothesize that mitochondrial dysfunction might be anchored in the genetic background, explaining decreased mitochondrial metabolism in both, iPNs and NPCs, leading to a higher energy supply by glycolysis, and as a consequence reduced glycolytic reserve in NPCs derived from MS. The findings that in proliferative cells, the energy is mainly obtained from glycolysis and is less dependent on mitochondrial metabolism (40, 53, 54), supports our findings that no difference in ERMCs was observed in NPCs differentiated from MS compared to HCs. Alterations of mitochondrial morphology in NPCs derived from MS might also be an compensatory mechanism by eliminating dysfunctional mitochondria followed by the generation of functional mitochondria causing a more fragmented morphology in NPCs derived from people with MS, as mitochondrial fragmentation is indicative for a higher mitochondrial density (50, 55, 56). Further, elevated mitochondrial mitophagy markers and higher parkin levels have been found in sera and CSF of people with MS. (57, 58) Possibly, by the metabolically shift from glycolysis to oxPhos during differentiation, mitochondria are still dysfunctional causing an energy deficit in MS-derived iPNs, demonstrated by decreased metabolism of both, glycolysis and oxPhos. In line, an impairment of the pyruvate metabolism was also observed in blood samples and brain tissue of MS. (59, 60) This impairment may lead to less byproducts, necessary for the function of oxPhos. However, it is possible that glycolysis is downregulated in MS-derived iPNs as a compensatory mechanism to generate more byproducts for oxPhos via metabolizing fatty acids or glutamine, as it was shown that glutamine levels were decreased in cerebrospinal fluid (CSF) and in the brain of MS. (61, 62)

Considering the controversial findings of mitochondrial morphology in iPNs compared to NPCs, it is likely that dysfunctional mitochondria might accumulate in the cell due to a possible lack of mitochondrial autophagy (63, 64), causing a more fused, elongated or swollen morphology (39, 50). Furthermore, the presence of fused mitochondria and expanded ERMCs in iPNs differentiated from people with MS can be also interpreted as a compensatory or stress-driven mechanism (65), reflecting an effort to optimize lipid transfer, calcium signaling, and cristae-structure organization (e.g., by increasing mitochondrial area, volume) to extract as much energy as possible (56, 66). This is also supported by findings that ERMCs are crucial for regulating mitochondrial metabolism (35, 67–70). Once this stage is chronic and excessive, it contributes to mitochondrial crisis and neuronal loss as heightened ERMCs may amplify oxidative stress and permeability of transition pore opening, despite transient gains in local energy supply (71–73). Conceptually, ERMC upregulation in MS-derived neurons therefore sits at the interface between metabolic compensation and degeneration: a morpho-functional adjustment that may sustain energy and ion homeostasis in the short term, but that, when chronic and excessive, accelerates mitochondrial dysfunction and neuronal vulnerability (74). Furthermore, ultrastructural studies in MS show that abnormal mitochondrial morphology and swollen mitochondria are one of the earliest signs of axonal damage preceding structural degeneration (24, 75). In addition, in another study there is also evidence that hyperfusion protects mitochondria from mitophagy/autophagy and from loss of respiratory chain activity (76, 77). On the other hand, it has also been shown that the process of necroptosis, a specific type of induced necrosis, occurs in cortical lesions and leads to oligodendrocyte degeneration in MS, which is also associated with mitochondrial swelling (78). Swollen mitochondria have been observed in early stages of ROS-induced axonal mitochondrial dysfunction (79). Mitochondrial swelling has also been observed in acute, chronic, and long-term chronic stages of EAE in mice (80).

### 4.1 Limitations

An important limitation of this study is the small number of biologically independent donor-derived cell lines investigated (MS: *N* = 3; healthy controls: *N* = 2). Consequently, the present data do not allow us to conclusively distinguish an MS-associated phenotype from donor-specific variation. The generation of these cell lines is technically demanding, and stable cell lines could be established from only three of 21 collected patient samples. Nevertheless, the observed effects were reproducible across multiple independent experiments performed on different days and using different cell passages. The present findings should therefore be regarded as proof of concept and require validation in a larger, independent donor cohort. In addition, cell size was not determined when assessing mitochondrial morphological parameters, and cell viability was not evaluated during the Seahorse experiments. These methodological limitations should be considered when interpreting the results. Moreover, our work is based on human-induced primary neurons, which do not represent a functional organism. Therefore, our results of the neuronal model whose significant results might not reflect the actual biological and physiological effect size, but could indicate possible genetic deficiencies, as the genetic information remains after reprogramming (81). Nevertheless, we cannot rule out possible mitochondrial mutations occurring during the differentiation process.

## 5 Conclusion

In conclusion, this study highlighted possible pre-existing, genetically determined altered mitochondrial and glycolytic functionality in human iPNs, supporting the findings of mitochondrial dysfunction (36–39), as well as an impaired glycolysis, possibly caused by impaired glucose and pyruvate metabolism in MS (59, 60). Furthermore, we observed increased ERMCs in MS-derived iPNs compared to HC-derived iPNs and a more fused mitochondrial morphology, supporting other studies of mitochondrial swelling in acute, chronic, and long-term stages of EAE (80). In addition a more fragmented mitochondrial morphology was observed in MS-derived NPCs, assuming that dysfunctional mitochondria are eliminated to maintain a functional mitochondrial network (55) (Figure 8). To the overall findings, the cell culture model used in this study also showed similar results in diseased humans such as decreased OCR and ECAR in PBMCs, T cells and endothelial cells from MS (61, 62, 82–84), highlighting its importance as a potential humanized model for MS research. Furthermore, by using this iPSC-based neuronal model we could demonstrate that a possible genetic predisposition for mitochondria plays a crucial role in MS. Since iPSC reprogramming erases acquired epigenetic modifications and environmental influences, the mitochondrial phenotypes observed in our MS-derived neurons are best interpreted as a reflection of the genetically encoded mitochondrial vulnerability contributing to MS pathogenesis.

**Figure 8:**
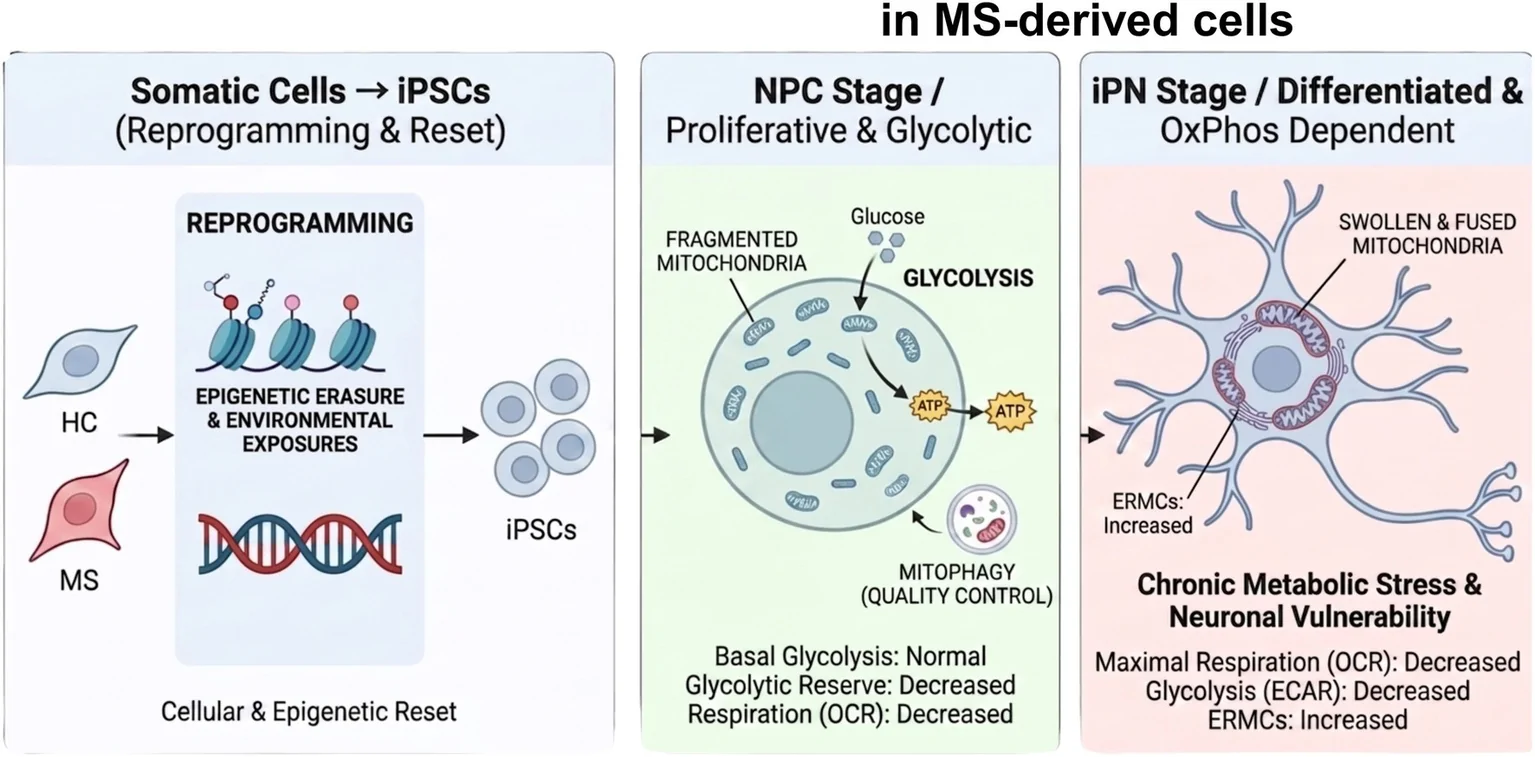
Summarized illustration of the study. Left panel describes the methodological procedure performed for the current study. Somatic cells were reprogrammed to indued pluripotent stem cells and differentiated to neuronal progenitor cells and induced primary neurons of healthy controls and people with Multiple Sclerosis. Mid panel highlights the results of a decreased mitochondrial metabolism, normal basal glycolytic metabolism, and fragmented mitochondrial morphology in MS-derived NPCs. Right panel addresses reduced mitochondrial and glycolytic metabolism as well as increased endoplasmic reticulum-mitochondria contacts and a fused mitochondrial morphology (Generated by bioRender AI, modified and based on our discussion part).

## Data Availability

The original contributions presented in the study are included in the article/Supplementary material, further inquiries can be directed to the corresponding author/s.

## Supplementary material

SUPPLEMENTARY TABLE 1 Information about patients included in this study. In total, 3 people diagnosed with Multiple sclerosis were included in this study (2 males and 1 female). The age ranged from 32 to 35 with a disease duration of 5-15 years. Patient 1 received a disease-modifying therapy with Dimethyl fumarate, patient 2 with Natalizumab and patient 3 with Fingolimod. The ratio of EDSS at the time point the urine was collected was 2.0-3.0.

SUPPLEMENTARY TABLE 2 Pilot experiment of assessing cell viability for Seahorse experiments. Three independent experiments including MS- and HC-derived cells were performed to assess cell viability during the Seahorse experiments. In each experiment 45 wells in total underwent Hoechst staining and measurement. The cell count varied between 91.495 to 112.648. The measurement was conducted after an real time experiment and it cannot be excluded that the cells died during the experimental period.

SUPPLEMENTARY FIGURE 1 Three-layer replication structure. First layer describes the biological replicates, as 3 MS-derived cell lines and 2 HC-derived cell lines were used. Second layer highlights the experimental repeats used for statistical analysis. Third layer explains the measurement of the mean of each triplicate in each experiment that was performed. For statistical analysis the number of experiments was used by taken together the mean of the triplicates of each MS-derived or HC-derived cell line in each experiment. (Figure was created by AI).

## Glossary

ATP: Adenosine triphosphate
Avg: Average
BDNF: Brain-Derived Neurotrophic Factor
CNS: Central Nervous System
CSF: Cerebrospinal Fluid
DG: 2-Desoxy-D-Glucose
DMEM/F12: Dulbecco’s Modified Eagle Medium F12 DMEM F12
EAE: Experimental Autoimmune Encephalitis
ECAR: Extracellular Acidification Rate
EDSS: Expanded Disability Status Scale
ER: Endoplasmic Reticulum
ERMC: Endoplasmic Reticulum-Mitochondria Contacts
FCCP: Carbonyl Cyanite-4 (Trifluoromethoxy) Phenylhydrazone
GDNF: Glia-Derived Neurotrophic Factor
HC: Healthy Control
iPN: Induced Primary Neuron
iPSC: Induced Pluripotent Stem Cell
mpH: Milli pH Units
MS: Multiple Sclerosis
mtDNA: Mitochondrial Deoxyribonucleic Acid
NADPH: Nicotinamide Adenine Dinucleotide Phosphate
NPC: Neuronal Precursor
NSC: Neuronal Stem Cell
OCR: Oxygen Consumption Rate
oxPhos: Oxidative Phosphorylation
PBMC: Peripheral Blood Mononuclear Cell
ROS: Reactive Oxygen Species
RPTEC: Renal Proximal Tubule Epithelial Cell
